# Transforming Growth Factor-Beta (TGF-***β***) Signaling in Paravertebral Muscles in Juvenile and Adolescent Idiopathic Scoliosis

**DOI:** 10.1155/2014/594287

**Published:** 2014-09-15

**Authors:** Roman Nowak, Magdalena Kwiecien, Magdalena Tkacz, Urszula Mazurek

**Affiliations:** ^1^Department of Orthopedics, School of Medicine with the Division of Dentistry, Medical University of Silesia, Wojewódzki Szpital Specjalistyczny nr 5 Plac Medyków 1, 41-200 Sosnowiec, Poland; ^2^Department of Molecular Biology, Medical University of Silesia, Ulica Narcyzów 1, 41-100 Sosnowiec, Poland; ^3^Institute of Computer Science, Division of Information Systems, University of Silesia, Ulica Będzińska 39, 41-200 Sosnowiec, Poland

## Abstract

Most researchers agree that idiopathic scoliosis (IS) is a multifactorial disease influenced by complex genetic and environmental factors. The onset of the spinal deformity that determines the natural course of the disease, usually occurs in the juvenile or adolescent period. Transforming growth factors *β* (TGF-*β*s) and their receptors, TGFBRs, may be considered as candidate genes related to IS susceptibility and natural history. This study explores the transcriptional profile of TGF-*β*s, TGFBRs, and TGF-*β* responsive genes in the paravertebral muscles of patients with juvenile and adolescent idiopathic scoliosis (JIS and AIS, resp.). Muscle specimens were harvested intraoperatively and grouped according to the side of the curve and the age of scoliosis onset. The results of microarray and qRT-PCR analysis confirmed significantly higher transcript abundances of TGF-*β*2, TGF-*β*3, and TGFBR2 in samples from the curve concavity of AIS patients, suggesting a difference in TGF-*β* signaling in the pathogenesis of juvenile and adolescent curves. Analysis of TGF-*β* responsive genes in the transcriptomes of patients with AIS suggested overrepresentation of the genes localized in the extracellular region of curve concavity: LTBP3, LTBP4, ITGB4, and ITGB5. This finding suggests the extracellular region of paravertebral muscles as an interesting target for future molecular research into AIS pathogenesis.

## 1. Introduction

Scoliosis is the most common spinal deformity in humans. Changes in spinal shape are three-dimensional and can be described as lateral curvature in the frontal plane, thoracic lordosis in the sagittal plane, and axial rotation in the horizontal plane. About 20% of scoliosis represent a phenotypic expression accompanying various pathologic conditions originating in almost every human tissue. The remaining 80% are idiopathic curves. Although the etiopathogenesis of idiopathic scoliosis (IS) is still unknown, there is an agreement about the multifactorial nature of this disorder [1–4]. A genetic model with two sets of genes, one responsible for the initiation and the other for the curve progression, well illustrates the multifactorial nature of IS. These genes could act separately or interact under the influence of diverse environmental and epigenetic factors [3, 5, 6]. The result of these complex interactions is the high heterogeneity of morphologic types, differences in the progression potential and the ages at presentation of the idiopathic curves. Paravertebral muscles play an essential role in the control of spinal stability [7, 8]. Imbalance of the paravertebral muscles could lead to biomechanical instability resulting in the development of a scoliotic curve. In addition, differences in progression among individuals may stem from divergence in muscle activation strategies or an inherent deficiency in structure and function of these muscles [9]. The Transforming growth factor-*β* (TGF-*β*) superfamily consists of a variety of cytokines expressed in many different tissues including skeletal muscles [10]. Expression of these molecules is linked to normal processes such as growth, differentiation, regeneration, and the stress response. TGF-*β* signaling is also required for cell regulation, angiogenesis, apoptosis, migration, extracellular matrix (ECM) remodeling and embryonic development [10–12]. TGF-*β*1 is one of the candidate genes in osteoporosis, and decreased bone mineral density (BMD) was observed in 27–68% of children with IS [13–18]. Osteopenia has also been suggested as one of the progression risk factors of IS [19, 20]. TGF-*β*1–3 isoforms are encoded by three different genes located respectively on chromosomes 19q13, 1q41, and 14q24 [11, 12]. Activated dimerized TGF-*β* proteins bind to cell-surface receptors type 1 and 2 (TGFBR1 and TGFBR2); transmembrane serine/threonine specific kinases that interact and phosphorylate intracellular molecules [21]. TGF-*β* ligands can also interact with the coreceptors endoglin and betaglycan, known as TGFBR3 [11]. Both endoglin and betaglycan can present TGF-*β*s to the TGFBR2 which then complexes with and phosphorylates TGFBR1 [21]. TGFBR3 is especially important for the TGF-*β*2 isoform, which, because of its low affinity, requires the presence of TGFBR3 to form a complex with TGFBR2 [22, 23]. Intracellular signaling of TGF-*β*s is mediated by proteins of Smads family. Smads 2 and 3 are substrates for receptors activated by TGF-*β*s and activins. Smads 1, 5, and 8 are downstream effectors for receptors activated by BMPs, GDFs, and MIFs. Phosphorylated Smads 1, 2, 3, 5, and 8 associate with Smad 4 and these complexes translocate to the nucleus, where together with other transcription factors they regulate the transcription of target genes [11, 24]. In addition to the canonical Smad pathway TGF-*β*s also signal through noncanonical pathways including mitogen activated kinase (MAPK), nuclear factor *κ*-B (NF-*κ*-B), Rho-like GTPase, phosphoinositide 3-kinase (PI3K)/Akt, hypoxia/hypoxia-inducible factor-1 (HIF-1) pathways [10, 11, 25]. Abnormalities in TGF-*β* signaling play an important role in various inherited and acquired musculoskeletal disorders where scoliotic deformity of the axial skeleton is one of the important clinical features. Progressive scoliosis occurs in approximately 45 to 60% of patients with Marfan syndrome (MFS) [26]. MFS is an autosomal dominant systemic disorder of connective tissue, caused by mutation of the FBN1 gene, which encodes extracellular matrix protein fibrillin-1 [10]. Fibrillin-1 stabilizes the Latent TGF-*β* complexes in the extracellular matrix. The various symptoms of Marfan syndrome are considered to be the result of an overall abnormality in the homeostasis of the extracellular matrix in which mutated forms of fibrillin-1 have led to alterations in mechanical properties of tissues, increased TGF-*β* signaling, and loss of appropriate cell-matrix interactions [24]. Marfan-like phenotypes may be also caused by mutations in TGF-*β* receptors. Scoliosis can be found in 46% of patients with Loeys-Dietz syndrome; a multisystem disease caused by a mutation in the genes encoding TGFBR1 or TGFBR2 [27]. Mutations of the gene encoding the TGF-*β*1 protein cause Camurati-Engelmann disease, which is associated with marfanoid habitus and increased TGF-*β* signaling, despite the absence of connective tissue fragility [28]. Increased TGF-*β* activity is also involved in the pathogenesis of muscular dystrophies characterized by variable progressive muscle weakness and wasting. In the most common form, Duchenne muscular dystrophy (DMD) mutations in the dystrophin gene lead to the loss of protection from contraction-induced injury. The resulting myocyte necrosis increases TGF-*β* signaling, which promotes muscle fibrosis at the expense of regeneration by satellite cells [29, 30]. Patients with DMD have a 90% chance of developing significant progressive scoliosis [31]. Despite many decades of multidisciplinary research, the cause(s) of IS remains unknown. The rapidly evolving arsenal of diagnostic and research tools offered by contemporary science allowed us to not only investigate 3-D deformity of IS not only through the radiological prism to determine changes in the spinal shape, but also to make an attempt at evaluating IS at the molecular level. In the last decade the number of studies investigating the molecular basis of IS has greatly increased. Much of the research work has focused on the identification of candidate genes related to connective tissue structure, bone formation and metabolism, melatonin signaling, puberty and growth, and axon guidance pathways [32]. Recently, in a case-control study, functional polymorphisms of the TGF-*β*1 gene were reported to be significantly associated with AIS susceptibility. In the female population, TGF-*β*1 polymorphism −509C > T also appeared to be associated with the age of disease onset and curve severity [33]. Most of idiopathic scoliotic curves are diagnosed during the juvenile or adolescent period [34]. The age of scoliosis onset determines its epidemiology, natural course and response to the treatment to a large extent [35–38]. Juvenile curves represent 12–21% of all scoliosis with unknown etiology [17, 35, 36, 39]. Curve morphologies are similar in both types of scoliosis, with the predomination of right primary thoracic and double primary thoracolumbar curves [37, 39]. The risk of deformity progression is the most important factor discriminating the juvenile type from the adolescent type of scoliosis. Patients with juvenile curves more often present with severe progression and 27% to 80% require operative treatment. In AIS, the risk of progression is much lower and only 0,1% of patients are operated on [35–38, 40, 41]. The reason(s) for different ages of scoliosis onset and associated differences in natural history are unknown. The results of a heritability study of 69 extended families in Utah with a history of AIS show that the onset of AIS is inherited separately from curve pattern and severity [42]. It also seems that genetic markers of progression in the adolescent type do not apply to idiopathic scoliosis with an early onset, below 9 years of age [43]. IS is presumed to be a multifactorial disease that is influenced by complex genetic and environmental factors. It is likely that curves with an earlier onset may have a different genetic background to curves appearing in adolescence. TGF-*β*s and their receptors may be included in the group of candidate genes involved in the etiology and pathogenesis of IS. Changes in the transcriptional profile of TGF-*β*s and their receptors (TGFBRs) could affect the expression of TGF-*β* responsive genes [44]. This could influence the regulation of a variety of signal transduction pathways potentially involved in IS etiopathogenesis. This involvement of TGF-*β* signaling could differ in juvenile and adolescent types of IS. Although TGF-*β*s and their receptors may be considered as candidate genes potentially related to IS susceptibility and natural history, so far nothing is known about the expression of these genes in the muscular tissue of IS patients. Therefore, the main aims of this study were.

1. Evaluation of the transcriptional activity of TGF-*β*1, TGF-*β*2, and TGF-*β*3 and their receptors TGFBR1, TGFBR2, and TGFBR3, in paravertebral muscles on both sides of the scoliotic curve in JIS and AIS.
2. Identification of the TGF-*β* responsive genes differentiating between the concave and convex sides of the scoliotic curve in juvenile and adolescent idiopathic scoliosis.

## 2. Materials and Methods

### 2.1. Material

The study design was approved by the Bioethical Committee Board of Silesian Medical University. Informed, written consent was obtained from each patient participating in the study and if required from their parents. Nine female patients average age 17.9 (13.4 to 25 years old), with a definite diagnosis of IS were included in the study. Five of the girls had a scoliotic curve diagnosed before the age of 10, and were designated as group A—juvenile idiopathic scoliosis, and four after 10 years of age, designated as group B—Adolescent Idiopathic Scoliosis. All of the patients had undergone posterior corrective surgery by the C-D method. According to the Lenke classification two curves were of type 2, four curves of type 3, two curves of type 5, one of type 6 [45]. Preoperatively, the average frontal and sagittal Cobb angles measured on standard p-a, and lateral radiograms were 67.3° (range 36°–94°) and 37° (range 20°–55°), respectively. Axial plane deformity was measured on computed tomography (CT) scans performed at the curve apex by spinal rotation angle relative to the sagittal plane (RAsag) and rib hump index (RHi), as described by Aaro and Dahlborn [46]. The mean RAsag was 19° (range 2.5°–36°) and RHi was 0.41 (range 0.03–0.74). Eighteen paravertebral tissue samples were collected intraoperatively; 9 from the concave (M1) and 9 from the convex (M2) side of the curve apex. One sample from concave and one sample from convex side of the curve were lost for further investigation in each of the studied groups (4 samples). Details of the participants in each of the studied group are listed in Table 1.

### 2.2. Molecular Analysis

Fourteen total RNA samples were submitted for gene expression analysis to determine the expression of TGF-*β* isoforms and their receptors TGFBRs with the use of HGU 133A oligonucleotide microarrays (Affymetrix) and qRT-PCR. Microarray data were further analyzed to identify TGF-*β* responsive genes differentially expressed between the concave and the convex sides of the curve in group A-JIS and group B-AIS. Extraction of total RNA from paravertebral muscles tissue samples was performed as described in a previous study [47]. Total RNA served as a matrix for microarray and qRT-PCR analysis of 14 transcriptomes. Muscular tissue samples preparation and HGU 133A microarray processing was performed according to Affymetrix Gene Expression Analysis Technical Manual. Details of the microarray processing were described previously [47]. Every step of the paravertebral muscle transcriptome processing method was verified qualitatively by electrophoresis on a 1% agarose gel stained with ethidium bromide and quantitatively by absorbance at 260 nm using a Gene Quant II spectrophotometer (Pharmacia LKB Biochrom Ltd.). After hybridization, fluorescence intensity was measured with an Agilent Gene Array Scanner G2500A (Affymetrix). After the microarrays were deemed suitable for comparative analysis, the fluorescence intensity values of all 22 843 transcripts of the 14 HG U133A chips were simultaneously normalized with the use of robust multichip average (RMA) algorithm [48]. The first step of the microarray analysis relied on a comparison of the fluorescence signals intensities of 10 mRNA IDs complementary to HGU 133A microarray probes between the paravertebral muscles of the concave and the convex side of the curve in JIS and AIS: three for TGF-*β*2, two for TGF-*β*1, two for TGFBR2, and one for each of TGF-*β*3, TGFBR1, and TGFBR3. The results of the microarray experiment were validated by qRT-PCR. Total RNA extracted from 14 paravertebral tissue samples served as a matrix for qRT-PCR of TGF-*β*1, TGF-*β*2, TGF-*β*3 mRNA and that of their receptors: TGFBR1, TGFBR2, and TGFBR3. Glyceraldehyde-3-phosphate dehydrogenase (GAPDH) and *β*-actin (ACTB) mRNA served as endogenous controls. The quantitative analysis was carried out with the use of a Sequence Detector ABI PRISM 7000 (Applied Biosystems). A standard curve was constructed for standards of ACTB (TaqMan DNA Template Reagents Kit, Applied Biosystems) and mRNA abundance in all tissue specimens was expressed as mRNA copy number per 1 *μ*g of total RNA. Details of the qRT-PCR method and the sequence of PCR primers were described in previous studies [49, 50].

Statistical analysis of the microarray results was performed with a GeneSpring 11 GX application (Agilent Technologies). Statistical analysis of the qRT-PCR results was performed with Statistica Version 10 software (StatSoft). Quantitative data were compared using a two-way ANOVA, and a nonparametric Mann Whitney *U* test, and *P* < 0.05 was considered statistically significant. Selected differentiating genes were classified by their gene ontology (GO) and analyzed statistically with the use of an overrepresentation test in a web-based, open access PANTHER database [51].

The microarray data from this experiment are publically available in MIAME-compliant format from the following address: http://www.ebi.ac.uk/arrayexpress/, the file ID is E-MTAB-980.

## 3. Results

Paravertebral muscles transcriptomes were grouped according to the side of the curve (M1—curve concavity, M2—curve convexity) and the age of scoliosis onset (A-juvenile idiopathic scoliosis, B-adolescent idiopathic scoliosis). Differentiation of the transcriptomes was evaluated in three steps. In the first step, TGF-*β*s and TGFBRs mRNA IDs were differentiated by oligonucleotide microarray chips HGU 133A (Affymetrix). In the second step, the microarray results were validated of the by qRT-PCR. Finally, 1050 mRNA IDs of 530 genes connected with TGF-*β* biological activity were selected based on Affymetrix data, and analysis of the expression profile of their transcriptomes was performed with the microarray data in order to select candidate genes differentiating paravertebral muscles from curve concavity and convexity in JIS and AIS.

### 3.1. Evaluation of TGF-*β*s and TGFBRs Expression in the Paravertebral Muscles of JIS and AIS Patients Using HG U133A Microarrays

**Figure 1:**
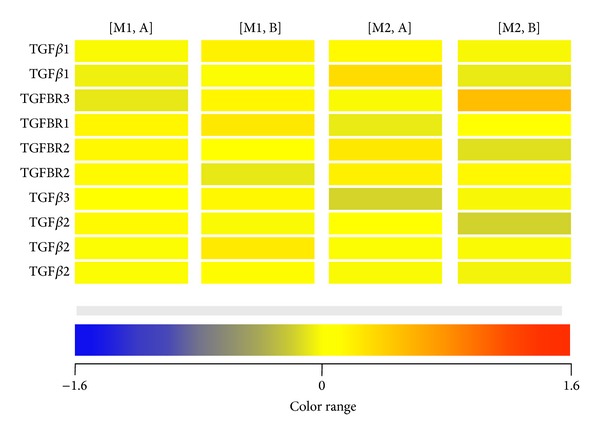
Heat map of fluorescence signal intensities of TGFB 1–3 and TGFBRs 1–3. Heat map of fluorescence signals intensities of 10 mRNA IDs of genes encoding TGF-*β* 1–3 and their receptors TGFBR 1–3 in paravertebral muscles of curve concavity (M1) and curve convexity (M2) in a group of juvenile (A) and adolescent idiopathic scoliosis (B).

Preliminary assessment of the mRNA fluorescence signal dispersion of the analyzed transcripts was performed with a heat map generated in GeneSpring 11 GX application. The heat map displays the normalized signal values, permitting the comparison of the transcriptional activity of the TGF-*β*s and TGFBRs in relation to the average transcriptional activity of paravertebral muscles. The expression value of each gene is mapped to a color-intensity value. Yellow indicates an average level of specific mRNA abundance in the analyzed transcriptomes. An increase in the mRNA abundance compared to the average value correlates with a change towards the red colors and decrease with a change towards the blue. Differentiation of the colors seen on the generated heat map indicates that transcriptional profile of the analyzed samples changes with the age of scoliosis onset (group A and B) and the side of the scoliotic curve (M1 and M2), as shown in Figure 1. Subsequent fold change analysis of differences in expression between the analyzed mRNA groups was performed with a nonparametric *U* Mann-Whitney test with permutative *P*-value computation. A fold change with a cutoff ≥ 1.1 and *P* < 0.05 was assumed to be statistically significant (Table 2).

Statistically significant differences in fluorescence signal intensity between the concave and convex paravertebral muscle transcriptomes were found for TGF-*β*2, TGF-*β*3 and TGFBR2 mRNA probes only in group B-AIS.

### 3.2. qRT-PCR Evaluation of the Transcriptional Profile of TGF-*β*s and TGFBRs in Paravertebral Muscles of JIS and AIS Patients

**Figure 2:**
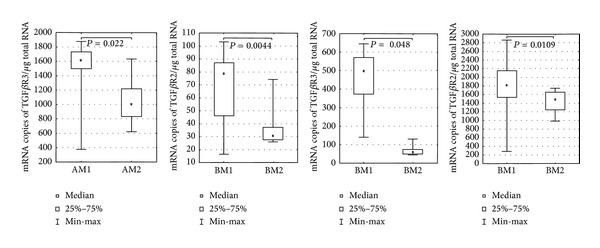
Evaluation of the transcriptional profile of TGF-*β*s and their receptors TGFBRs in paravertebral muscles of JIS group A and AIS group B by qRT-PCR. Box and whisker plots illustrating statistically significant (nonparametric *U* Mann-Whitney test, *P* < 0.05) results of the comparison of the mRNA abundance of TGF-*β*s and TGFBRs between concave and convex sides of the curve in both analyzed groups. AM1, AM2, respectively, paravertebral muscle of concave and convex side of the curve in JIS group and BM1, BM2 samples from concave and convex side of the curve in AIS group.

qRT-PCR was used to evaluate the transcriptional profile of TGF-*β*1–3 and their receptors, TGFBR1–3 in the paravertebral muscles of patients with JIS and AIS. The abundance of TGF-*β*s and TGFBRs mRNA in the concave (M1) and convex (M2) sides of the curve in groups A and B confirmed the results of the HGU 133A microarray experiment. Significantly higher abundances of TGF-*β*2, TGF-*β*3 and TGFBR2 mRNA (nonparametric *U* Mann-Whitney test, *P* < 0.05) on the concave side of the curve were found in group B. In group A, a statistically significant difference in the abundance of TGFBR3 mRNA was found, however, this was not confirmed by the preceding microarray experiment (Table 3, Figure 2).

### 3.3. Transcriptional Profile of TGF-*β* Responsive Genes

**Figure 3:**
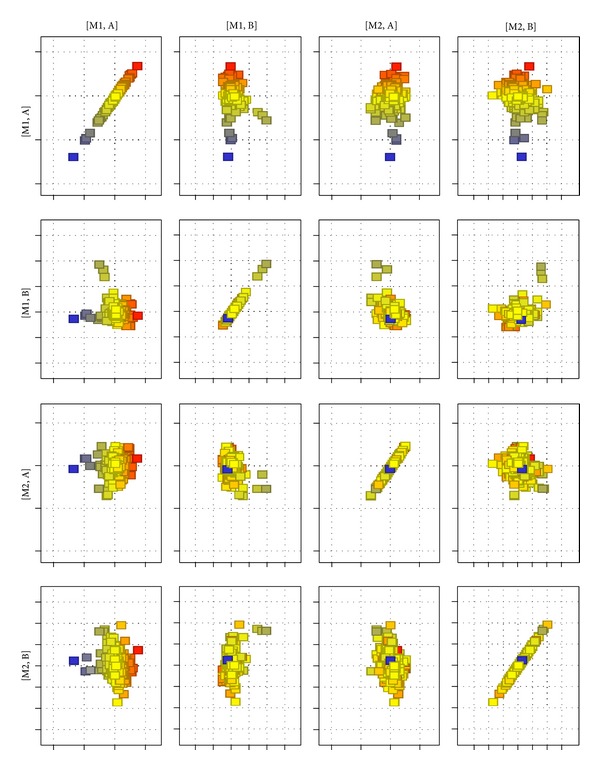
TGF-*β* responsive genes in the paravertebral muscles of JIS and AIS patients. Matrix plot illustrating the degree of differentiation of 1050 mRNA IDs of TGF-*β* responsive genes between the transcriptomes of muscular tissue in dependence of the side of the curve (M1 versus M2) and the age of scoliosis onset (A versus B). Red spots upregulated genes, blue spots downregulated genes. M1, M2: respectively muscular tissue samples from curve concavity and convexity, A: juvenile idiopathic scoliosis; B: adolescent idiopathic scoliosis.

Differences in the expression of TGF-*β* responsive genes between the paravertebral muscles of the concave and convex sides of the curve were evaluated in groups A and B. 1050 mRNA IDs of TGF*β*-associated genes were selected from 22843 transcripts that could be analyzed with HGU 133A chips [52]. A matrix plot of normalized log-intensities was used to visualize the degree of difference in TGF-*β* responsive genes between muscular tissue transcriptomes harvested from curve concavity and convexity in both groups. The main purpose of the matrix plot is to obtain an overview of the correlation between conditions in the dataset and detect conditions that separate the data into different groups. Higher intersample differences are interpreted through the distance of the spots from the regression line. Matrix plot analysis of the expression data highlighted a greater number of up- and downregulated genes in the paravertebral muscles of group A (AM1 versus AM2) compared to group B, where these differences were less pronounced. The matrix plot generated also indicates greater differences in TGF-*β* responsive gene expression at the mRNA level between groups A and B in the specimens from the curve concavity (AM1 versus BM1). The difference was much less clear between the transcriptomes from the curve convexity (AM2 versus BM2—Figure 3).

**Figure 4:**
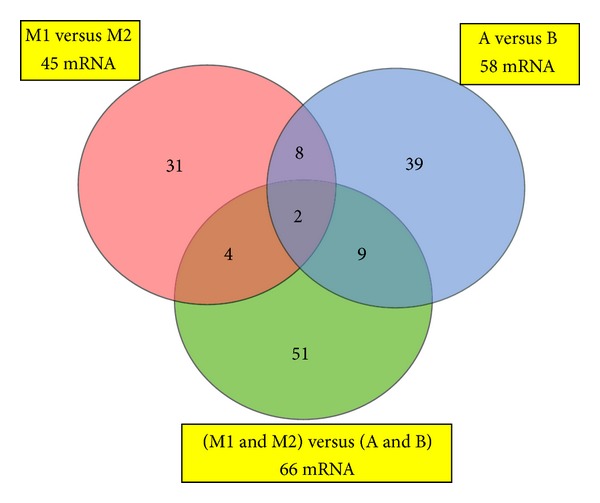
Venn diagram of two way ANOVA test for TGF-*β* responsive genes. Venn diagram illustrating the number of TGF-*β* responsive genes with a *P* value < 0.05 (two way ANOVA) differentially expressed between the sides of the curve (M1 versus M2), age of scoliosis onset (A versus B) and their interactions.

**Figure 5:**
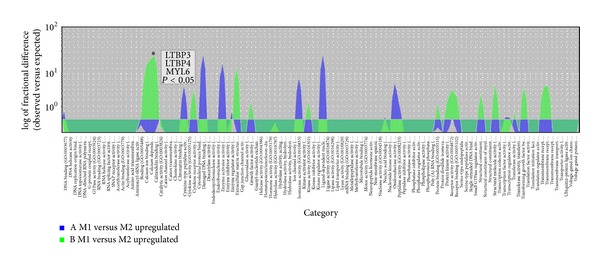
Overrepresentation test of upregulated genes differentiating muscular transcriptomes in JIS and AIS—GO molecular function. Overlaid area chart of difference presenting the results of an overrepresentation test of GO molecular function of upregulated genes differentiating concave and convex paravertebral muscle transcriptomes in JIS and AIS group. ∗ above the peak mark statistically significant result of overrepresentation test, *P* < 0.05 calculated with Bonferroni multiple correction.

**Figure 6:**
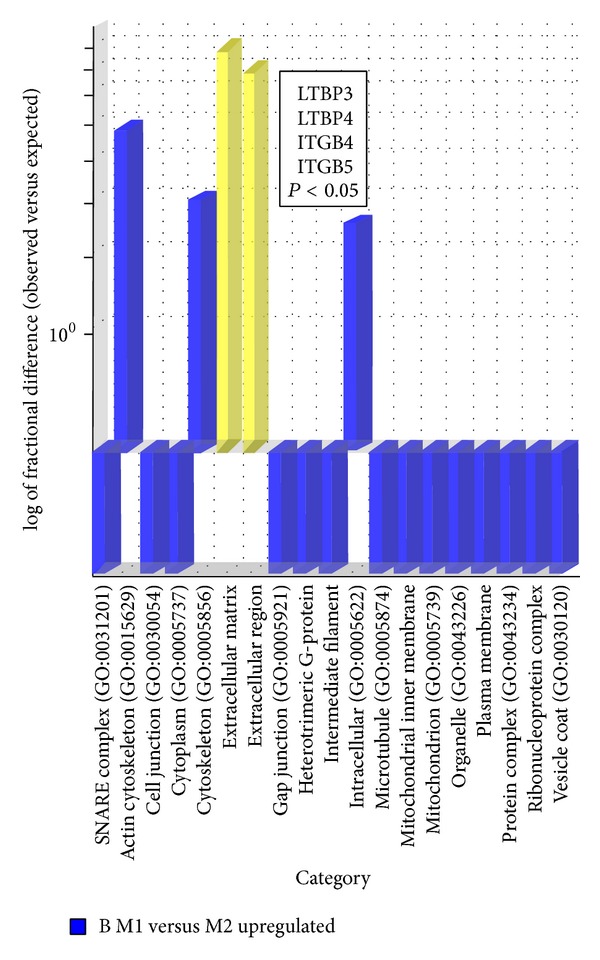
Overrepresentation test of upregulated genes differentiating muscular transcriptomes in AIS—GO cellular component localization. Bar chart of difference presenting the results of an overrepresentation test of GO cellular component localization of upregulated genes differentiating concave and convex paravertebral muscle transcriptomes in the AIS group. Yellow bars indicate statistically significant results of overrepresentation test, *P* < 0.05 calculated with Bonferroni multiple correction.

Further statistical analysis of the microarray data was performed by means of two-way ANOVA to test the main effects of the curve side (M1, M2), and age of scoliosis onset (A, B), and their interactions. Through this analysis, 144 genes were identified as having significantly different expression (*P* < 0.05) either with respect to the of the curve side, the age of scoliosis onset, or both. The Venn diagram in Figure 4 displays the numbers of these genes products whose *P* values were influenced by each parameter independently (side of the curve and age of scoliosis onset) and also by the effect that each of the parameters had on the other. From a total of 45 genes that displayed a curve side main effect, 31 were significant due to the curve side effect only, while 4 genes displayed the curve side effect and an interaction effect. Expression of another 58 genes displayed an age of scoliosis onset effect. Of those, 8 were affected by both side of the curve and age of onset, but each of the factors affected gene expression independently one of another. In the case of 51 genes that were statistically significant neither with the regard to the side of the curve, nor to the age of scoliosis onset, the two-way ANOVA test had a *P* value < 0.05 for the interaction effect. Nine genes were statistically significant for the age of onset effect and interaction effect but not the curve side effect. Four genes were statistically significant with regard to the side of the curve effect and interaction effect. Two genes displayed all the three effects: side of the curve, age of onset and interaction (Figure 4). Interaction effects indicate that there is no overall effect, but rather an effect that changes with the levels of another variable. As it is dangerous to interpret main effects when a gene shows an interaction effect, further statistical analyses were performed with nonparametric *U* Mann-Whitney test, with permutative *P*-value computation type (100 permutations). Fold change with a cutoff > 1 and *P* < 0.05 was assumed to be statistically significant. In consequence, a group of 44 mRNA IDs of TGF-*β* related genes differentially expressed between M1 and M2 paravertebral muscles was selected from group A and 34 mRNA IDs from group B. Further selection of differentially expressed genes was performed by comparison of the results of the two-way ANOVA and nonparametric *U* Mann-Whitney tests. Only those genes that fulfilled the criteria of statistical significance in both tests were considered for GO classification. A total of 24 genes (10 upregulated and 14 downregulated) differentiating paravertebral M1 and M2 transcriptomes were selected from group A and 11 genes (7 upregulated and 4 downregulated) from group B. Selected genes were subsequently classified by their GO molecular function through the open access PANTHER database (Table 4). Both lists of differentiating genes were subsequently divided into upregulated and downregulated groups and further analyzed statistically using an overrepresentation test; a binominal statistical tool that takes a list of genes and finds PANTHER functional classes that are overrepresented or underrepresented in the list compared with a selected reference list of genes [51]. Overrepresentation test analysis was performed in terms of GO molecular function, biological process and cellular component with the use of Bonferroni correction for multiple testing; *P* < 0.05 was considered statistically significant. The reference list comprised 1050 mRNA IDs of 530 TGF-*β* related genes that were analyzed with HGU 133A microarray chips. This analysis revealed no statistically significant results in group A. In addition, in group B no statistically significant results were found neither in the group of downregulated genes nor in the group of upregulated genes in the GO category of biological process. However analysis of GO molecular function in the group of genes upregulated in group B showed statistically significant overrepresentation of genes connected with calmodulin binding (LTBP3, LTBP4, and MYL6; Figure 5). Analysis of the GO cellular component localization of the upregulated genes of the group B indicated statistically significant overrepresentation of genes localized in the extracellular matrix and extracellular region (LTBP3, LTBP4, ITGB4, and ITGB5), (Figure 6).

## 4. Discussion

In two previous studies significantly higher expression of TGF-*β*1 was found on the concave side of the curve in the vertebral cartilaginous endplates and articular cartilages of the apical articular processes of AIS patients, suggesting that TGF-*β*1 is involved either as an etiological factor or a secondary change in the curve development [53, 54]. In the first part of this work, gene expression profiling was used in order to identify differences in the expression of TGF-*β*s and their receptors, TGFBRs, between both sides of the curve in the paravertebral muscles of juvenile and adolescent idiopathic scoliosis. Significantly higher abundances of TGF-*β*2, TGF-*β*3, and TGFBR2 transcripts were confirmed using both microarray and qRT-PCR techniques in muscular tissue samples collected from the curve concavity of AIS patients. The expression pattern of TGF-*β*s and their receptors was not previously examined in the paravertebral muscles of IS patients with different ages of deformity onset. Increased transcriptional activity of TGF-*β*2, TGF-*β*3, and TGFBR2 seen on the concave side of the curve in AIS patients might suggest the involvement of TGF-*β* signaling in the pathogenesis of scoliotic curves with later onset. However it should be pointed out that all of the tissue samples were harvested from patients with severe curves, long after the deformity onset. The selection of patients with severe idiopathic scoliosis curves for experimental groups can be problematic because such patients represent the extreme cases, and at the time of the study they are usually much older than, when the curve started to develop [4]. The average age of the deformity onset (diagnosis) in the JIS group was 7.2 years old and in AIS group 12.5. The average age of patients at the time of the operative treatment, when the tissue specimens were collected, was 17.9 years old. Thus it is possible that increased TGF-*β* signaling at the curve concavity was also present in the JIS group but earlier in the curve evolution. Differences in TGF-*β*2, TGF-*β*3 and TGFBR2 expression between JIS and AIS may need definitive confirmation by histological staining. Despite the similarity of their actions* in vitro*, each of the TGF-*β* isoforms appears to mediate a different set of actions* in vivo*. Knocking out TGF-*β*1, TGF-*β*2, and TGF-*β*3 in mice has shown no phenotypic overlap, indicating that these isoforms are functionally noncompensated [21, 23, 55]. The difference in the expression of TGF-*β*2, TGF-*β*3 and TGFBR2 observed between the sides of the curve in the AIS group may be a secondary phenomenon in the scoliosis progression, reflecting an increase in the extent of fibrotic changes to the curve concavity; as TGF-*β*s are considered critical regulators of physiological fibrogenesis and pathological fibrosis. TGF-*β*1 and TGF-*β*2 are potent profibrotic factors, whereas TGF-*β*3 reduces the fibrotic response. It seems that TGF-*β*2 is secreted only at the initial stage of fibrosis as an assistant factor to TGF-*β*1. The ratio TGF-*β*1/TGF-*β*3 is also of importance and might influence the progression of the fibrotic response [12, 56]. Stimulating cells with TGF-*β*s immediately leads to positive and negative changes in the expression of several hundred genes. Many of these gene responses depend on the cell type and other conditions affecting the cell at the time of TGF-*β* stimulation [57, 58]. The differences in the expression of TGF-*β* isoforms and their receptors observed in this study between the concave and convex sides of the curve may suggest different role of TGF-*β* signaling in juvenile and adolescent idiopathic curves. This could be reflected by differences in the transactivation of TGF-*β* responsive genes. Thus, in the second part of this work we analyzed paravertebral muscle transcriptomes from JIS and AIS individuals in order to identify the TGF-*β* responsive genes that are differentially expressed between the concave and convex sides of the curve. Statistical analysis of the expression profile of 1050 mRNA probes of 530 TGF-*β* responsive genes permitted the selection of 10 upregulated and 14 downregulated candidate genes in JIS and 7 upregulated and 4 downregulated genes in AIS. There was no overlap between differentially expressed TGF-*β* related genes in the JIS and AIS groups. This observation supports the idea of differences in the involvement of TGF-*β* signaling in paravertebral muscles of IS patients with different ages of onset. The results of the overrepresentation test were statistically significant only in the AIS group of upregulated genes for the categories of GO molecular function and GO cellular component. In the category of GO molecular function statistically significant overrepresentation of genes involved in calmodulin binding was revealed: Myosin light polypeptide 6 (MYL6), latent transforming growth factor beta binding protein 3 (LTBP3), and latent transforming growth factor *β* binding protein 4 (LTBP4). The significant overexpression of genes involved in calmodulin binding seems to be interesting in the context of some previous studies concerning IS. It has been shown that an increased calmodulin concentration in platelets is associated with progression of AIS [59, 60]. The platelet calmodulin changes were attributed to paravertebral muscle activity, and a role for calmodulin in the etiopathogenesis of IS as a systemic mediator of tissues with contractile properties was suggested [60]. In animal models of IS administration of tamoxifen, a calmodulin antagonist, appeared to decrease the magnitude and incidence of the deformity [61, 62]. Examination of paravertebral muscles of patients undergoing surgery for AIS revealed higher concentrations of calmodulin in muscle specimens of the curve convexity [63]. Myosin light polypeptide 6 is a smooth muscle and nonmuscle myosin light chain encoded by the MYL6 gene, located on chromosome 12q13.2. MYL6 protein is one of the two essential light chains of the actin-based motor protein complex of myosin. The functional roles of the essential light myosin chains in the smooth muscle are not fully understood. It is likely that these proteins affect the interaction between the two myosin heads when the regulatory light chains are phosphorylated [64, 65]. It has been suggested that the type of the essential myosin light chain influences the maximal shortening velocity in smooth muscles [66]. Expression of MYL6 in human skeletal muscles has been confirmed by microarray investigations [67]. The role of MYL6 upregulation observed in the paravertebral muscles of the curve concavity in the pathogenesis of the scoliotic deformity in the AIS group remains to be elucidated. The protein products of TGF-*β*1–3 genes are secreted from cells to the extracellular space and maintained in an inactive form in a complex with latency associated polypeptide (LAP) and latent TGF-*β* binding proteins (LTBPs) [21]. LTBPs are large glycoproteins structurally related to fibrillin. Through interactions with divergent proteins, LTBPs affect the bioavailability of TGF-*β*s and play an important structural role in elastic fibril and microfibril organization and function [23, 66, 68]. Four different LTBPs are known, of which LTBP3 binds all three TGF-*β* LAP isoforms with high affinity, whereas LTBP4 shows a weak binding capacity only for TGF-*β*1 LAP [23, 69]. LTBP3 can also associate with a pro-form of myostatin, a TGF-*β*-like hormone that regulates the size of skeletal muscles [23]. Larger back muscle volume at the concave side of the curve apex in AIS patients has been confirmed by MRI data [70]. LTBP4 is highly expressed in skeletal muscle and has been postulated to be a determinant of damage and fibrosis in muscle diseases [71]. Studies in humans and mice show that LTBP4 performs a functional role in promoting elastogenesis and in regulating TGF-*β* activity [68]. Integrity of the structures stabilizing the spine, including the paravertebral muscles, depends to a large degree on the constituents of the extracellular matrix and their response to biomechanical load. Immunohistochemical studies on ligamentum flavum specimens from patients with AIS revealed pathological changes in the elastic fibers of the microfibrils [26]. Defects in the elastic fiber system may result in spinal imbalance and lead to spinal deformity. Elastic proteins have been observed in spinal connective tissues as well as in the muscle and bone matrix of scoliotic patients [72]. The upregulation of LTBP3 and LTBP4 seen in this study may suggest that these proteins, as important ECM components, could play an important role in the pathogenesis of AIS. This was further supported by GO cellular component overrepresentation test results. Statistically significant overrepresentation of genes localized in the ECM and extracellular region was found. In addition to LTBP3 and LTBP4, statistical analysis highlighted two other upregulated genes that were differentially expressed in the concave and convex sides of the curve in the AIS group: integrin beta4 (ITGB4), and integrin beta5 (ITGB5). The ECM is a complex three-dimensional network of macromolecules secreted and deposited into the space surrounding cells [28]. The ECM regulates cell behavior by acting as a substrate for cell migration, modulating growth factor activity, transmitting signals and serving as a structural framework necessary for normal structural integrity [24, 73]. Integrins are heterodimeric transmembrane receptors that attach cells to the surrounding ECM and mediate both cell-cell and cell-ECM interactions. These proteins play a role in bidirectional signaling across the cell membrane in order to regulate cell adhesion, migration, and proliferation as well as differentiation and extracellular matrix remodeling. Additionally, integrins can modulate the signaling pathways of many growth factors, including TGF-*β*s [74–78]. In humans, each of the 24 known integrins is composed of one of 18 alpha subunits and one of 8 beta subunits. Each subunit contains a large extracellular domain, a transmembrane region, and a cytoplasmic tail [77, 78]. The combination of subunits determines the specificity of integrins for ECM molecules [76, 79, 80]. The extracellular domains bind with ECM proteins such as fibronectin, laminin and collagen. The cytoplasmic domains of beta subunits interact with kinases such as focal adhesion kinase and Src kinase, adaptor molecules like talin and kindlin and the cytoskeleton (actin and microtubules) [77]. Integrins also act as mechanotransducers, which sense tension generated either by cytoskeletal elements or the ECM [75, 81, 82]. Such integrin-mediated mechanotransduction plays an important role in remodeling and functional adaptation of skeletal muscle tissue to ensure an optimal force transmission with muscle contraction [79, 82]. Integrin beta4 (ITGB4), and integrin beta5 (ITGB5) subunits are encoded by genes located respectively on chromosomes 17q25 and 3q21 [67]. ITGB4 tends to associate with integrin alpha6 subunit and is one of the highly selective receptors of laminin, a major structural component of the basement membranes of epithelial tissues [76, 83]. In contrast to the majority of integrins alpha6beta4 does not connect to the actin cytoskeleton but to the intermediate filament system [75, 83]. It appears that, besides its role in stable adhesion, integrin alpha6beta4 is upregulated in wound healing and can play the role of master regulator of the expression levels of the other integrins in the epidermis [84]. Recent studies show that in skeletal muscle, beta4 integrin marks interstitial progenitor cells that are distinct from satellite cells but exhibit myogenic potential [85, 86]. Integrin alphavbeta5 functions as a major vitronectin receptor and, to lesser extent, as a fibronectin receptor [76, 79]. TGF-*β*1 and TGF-*β*3 can be activated through their interaction with several integrins, including alphavbeta5 [74, 76, 78, 87]. Activation of TGF-*β* by alphavbeta5 could be important in pathological conditions, as illustrated by an increased expression of this integrin in the dermis of scleroderma patients [84]. Additionally, TGF-*β* activation by alphavbeta5 is important in pulmonary fibrosis [74]. Interestingly, alphavbeta5 is also a molecular marker for skeletal muscle mononuclear cells, including satellite and progenitor interstitial cells [79]. The upregulation of ITGB4 and ITGB5 observed in the paravertebral muscles of the curve concavity of AIS patients merits further investigation as this could lead to the disequilibrium between the two sides of the curve and could be involved in the pathogenesis of AIS. However caution should be taken when drawing definite conclusions about the role of ECM macromolecules in the evolution of scoliotic curves, due to the small number of specimens analyzed in this study and the lack of a control group. Our results may need validation in another population with larger sample size. The exact genetic mechanisms that contribute to spinal malformation in IS are still to be unraveled. Neuromuscular abnormalities, in conjunction with adverse mechanical environments, in which hormonal and other chemical factors act as regulators of skeletal muscle tone and function, are possible explanations for the pathogenesis of IS [88]. The interactions between different cell types of the neuromuscular system are mainly mediated by diffusible factors, many of which are growth factors like TGF-*β*s [89]. Further molecular research is needed to determine whether interactions between growth factor signaling pathways, such as the TGF-*β* pathway, and transcriptional regulatory networks lead to the initiation and progression of scoliotic deformities. At present, treatment of IS is focused on symptoms and involves bracing, and surgery in more severe cases. The identification of proteins and signaling pathways with significant asymmetry in expression pattern between concave and convex side of the curve could suggest new biomarkers of progression risk and aid in developing novel therapeutics to combat structural changes of the spine.

## 5. Conclusions

The transcriptional activity of TGF-*β*2, TGF-*β*3, and TGFBR2 and the expression profile of TGF-*β* responsive genes differ in paravertebral muscle transcriptomes depending on the age of scoliosis onset and the side of the scoliotic curve. This phenomenon could signify a different involvement of TGF-*β* signaling in the pathogenesis of juvenile and adolescent curves. Analysis of TGF-*β* responsive genes that differ in the concave and convex paravertebral muscle transcriptomes of AIS patients highlights the upregulation of genes localized in the extracellular region of the concave side of the curve (LTBP3, LTBP4, ITGB4, and ITGB5). This finding suggests that the extracellular region of paravertebral muscles is an interesting target for future molecular research on AIS pathogenesis.

## Figures and Tables

**Table 1:** Characteristics of patients of both studied groups. Group A: Juvenile idiopathic scoliosis, group B: adolescent idiopathic scoliosis. M1, M2 samples from curve concavity and convexity. RAsag: rotation angle sagittal, RHindex: rib hump index.

Patient	Sample	Group	Lenke type	Cobb angle	Kyphosis angle	RAsag	RHindex
K.P.	M2	A	3	68	33	14	0.48
F.J.	M1, M2	A	3	94	50	24	0.56
B.P.	M1	A	6	36	48	2.5	0.03
R.K.	M1, M2	A	2	60	34	17	0.36
N.M.	M1, M2	A	5	75	30	21.5	0.5
M.S.	M1	B	3	66	30	6	0.03
J.K.	M2	B	2	88	55	29	0.64
P.A.	M1, M2	B	5	42	40	21	0.3
W.P.	M1, M2	B	3	77	20	36	0.7

**Table 2:** Comparison of fluorescence signal intensity of mRNA IDs of TGFBs and TGFBRs in paravertebral muscle transcriptomes from curve concavity (M1) and convexity (M2) in JIS (group A) and AIS (group B) patients.

Group	ID	Symbol	NCBI-ID	Mann-Whitney	FC	Regulation
A (M1 versus M2)	203085_s_at	TGF-*β*1	7040	NS	1.3	Up
	204731_at	TGFBR3	7049	NS	1.1	Up
	206943_at	TGFBR1	7046	NS	1.1	Down
	209908_s_at	TGF-*β*2	7042	NS	1.1	Down

B (M1 versus M2)	207334_s_at	TGFBR2	7048	0.009	1.1	Up
	209747_at	TGF-*β*3	7043	0.014	1.1	Up
	209908_s_at	TGF-*β*2	7042	0.016	1.2	Up

**Table 3:** Comparison of the qRT-PCR transcriptional profile of TGFBs and TGFBRs in paravertebral muscles of the concave (M1) and convex (M2) sides of the curve in JIS (group A) and AIS (group B) patients.

Group	*P* value nonparametric *U* Mann-Whitney test					
	TGF-*β*1	TGF-*β*2	TGF-*β*3	TGFBR1	TGFBR2	TGFBR3
A (M1 versus M2)	0.492	0.958	0.313	0.562	0.683	0.022
B (M1 versus M2)	0.683	0.0044	0.048	0.157	0.0109	0.214

**Table 4:** GO molecular functions of TGF*β* related genes differentiating muscular transcriptomes from concave and convex sides of the curve in JIS: juvenile idiopathic scoliosis and AIS: adolescent idiopathic scoliosis. *P* value result of nonparametric *U* Mann-Whitney test. FC: fold change.

Function	JIS				AIS			
	Gene	*P*	FC	Regulation	Gene	*P*	FC	Regulation
Transcription factor activity	NR3C1	0.04	1.56	up	TOX4	0.01	1.17	down
	SMAD3	0.02	1.18	up				
	TRIM33	0.03	1.38	down				
	ZBTB7B	0.02	1.26	down				

Growth factor activity	BMP2K	0.04	1.12	down	GDF15	0.03	1.17	up
	BMP6	0.02	1.17	down				
	INHBA	0.01	1.18	down				

Receptor activity	TACD2	0.02	1.22	down	ITGB4	0.01	1.09	up
					ITGB5	0.01	1.15	up

Calcium ion binding	FKBP1A	0.01	1.37	up				
	PCDH1	0.01	1.3	down				

Calmodulin binding					LTBP3	0.03	2.34	up
					LTBP4	0.01	1.5	up
					MYL6	0.03	1.53	up

Protein binding	FBXL12	0.01	1.31	up				
	ASPP1	0.02	1.23	down				

Hormone activity					PTHR	0.02	1.1	down

Cysteine type peptidase activity	UCHL5	0.03	1.56	up				

Oxidoreductase activity	EGLN1	0.04	1.52	up				

Str. constituent of cytoskeleton	KRT15	0.01	1.26	down				

Ubiquitin protein ligase activity					SMURF1	0.03	1.04	down

Nucleotide kinase activity					MAGI2	0.02	1.2	down

Transmembrane receptor protein-kinase activity					TGFBR2	0.01	1.13	up

Unclassified	FA175	0.02	1.26	up				
	TTC17	0.04	1.21	up				
	WIPI3	0.02	1.19	up				
	TMEM1	0.03	1.1	up				
	UNC45	0.03	1.43	down				
	DCAF7	0.01	1.23	down				
	SH3D21	0.01	1.19	down				
	RAB25	0.01	1.13	down				
	L1TD1	0.02	1.05	down				
